# Supplementary material for: Constrained portfolio optimization with discrete variables: An algorithmic method based on dynamic programming
Source: PLoS One. 2022 Jul 28;17(7):e0271811. doi: 10.1371/journal.pone.0271811 (PMC9333297; doi:10.1371/journal.pone.0271811)
Supplement: S1 Appendix — (PDF) [file pone.0271811.s001.pdf]

```matlab
clc
clear
close all
format short

disp('----------------   test_lambdaDynamicprog.m is in process --------
------')

%%%%%%%%%%%%%%%%%%%%%%%%%%%%%%%%%%%%%%%%%%%%%%%%%%%%%%

name='Initial Data.xlsx';
sheet='Data';

%%%%%%%%%%%%%%%%%%%%%%%%%%%%%%%%%%%%%%%%%%%%%%%%%%%%%%

A=xlsread(name,sheet);
Lambda_star=A(1,1);
dimx=A(1,2);
Pi=A(:,4);
Ri=A(:,5);
lb=A(:,6);
ub=A(:,7);
B=A(1,8);
L=A(1,9);

N=numel(Pi);
vL=0:L:B;
vL(end)=B;

vlb=vL(1:end-1);
vub=vL(2:end);


%%%%   please set the intervsl %%%%%%%%%%%%%%%%% set the favour interval

Lambda_a=-2;
Lambda_b=2;
LL=Lambda_a:0.05:Lambda_b;

%%%%%%%%%%%%%%%%%%%%%%%%%%%%%%%%%%%%%%%%%%%%%%%%%%%%%%

MM=nan(6+dimx,numel(LL));
for ii=1:numel(LL)
    Lambda_star = LL(ii);

[Solution_a,Ra]=Dynamic_prog(Lambda_star,dimx,Pi,Ri,lb,ub,N,vlb,vub,'no-
print');
```

```
    snum=sum(Solution_a>0);
     MM(:,ii)=[LL(ii);nan;Ra;nan;Solution_a;nan;snum];
end

xlswrite(strcat(name ,'-Dynamic-Result-40up40.xlsx'),MM,'compare','c3')

disp('------------   test_lambdaDynamicprog.m was terminated
successfully ------------')
```
