# Supplementary material for: Constrained portfolio optimization with discrete variables: An algorithmic method based on dynamic programming
Source: PLoS One. 2022 Jul 28;17(7):e0271811. doi: 10.1371/journal.pone.0271811 (PMC9333297; doi:10.1371/journal.pone.0271811)
Supplement: S2 Appendix — (PDF) [file pone.0271811.s002.pdf]

```
clc
clear
close all
format short

disp('-----------------   test_lambdaintlinprog.m is in process ---------
-----')

%%%%%%%%%%%%%%%%%%%%%%%%%%%%%%%%%%%%%%%%%%%%%%%%%%%%%%

name='Initial Data.xlsx';
sheet='Data';

%%%%%%%%%%%%%%%%%%%%%%%%%%%%%%%%%%%%%%%%%%%%%%%%%%%%%%

A=xlsread(name,sheet);
Lambda_star=A(1,1);
dimx=A(1,2);

Pi=A(:,4);
Ri=A(:,5);
lb=A(:,6);
ub=A(:,7);
B=A(1,8);
N=numel(Pi);

%%%%   please set the intervsl %%%%%%%%%%%%%%%%%%%%%%%%%%%%%%%%%
% set the favour interval

Lambda_a=-10;
Lambda_b=10;
LL=Lambda_a:0.1:Lambda_b;

%%%%%%%%%%%%%%%%%%%%%%%%%%%%%%%%%%%%%%%%%%%%%%%%%%%%%%

A=[Pi' zeros(1,N);eye(N) -diag(ub);-eye(N) diag(lb)];
b=[B;zeros(2*N,1)];

Aeq=[];beq=[];
lb0=zeros(2*N,1);
ub0=[ub;ones(N,1)];


MM=nan(2*dimx+8,numel(LL));
for ii=1:numel(LL)
```

```
    Cc=[Ri;-LL(ii)*ones(N,1)];

    [Solution_a,Ra,Exitflag]=intlinprog(-
Cc,1:(2*N),A,b,Aeq,beq,lb0,ub0);

    MM(:,ii)=[LL(ii);nan;Exitflag;nan;Ra;nan;Solution_a];

end

xlswrite(strcat(name ,'-intlinprog-Result-
40up40.xlsx'),MM,'compare','c3')

disp('------------   test_lambdaintlinprog.m was terminated successfully
------------')
```
