# Supplementary material for: Constrained portfolio optimization with discrete variables: An algorithmic method based on dynamic programming
Source: PLoS One. 2022 Jul 28;17(7):e0271811. doi: 10.1371/journal.pone.0271811 (PMC9333297; doi:10.1371/journal.pone.0271811)
Supplement: S3 Appendix — (PDF) [file pone.0271811.s003.pdf]

Table 1: Information on the objective function and variables for different values of λ.

-1.55	-1.6	-1.65	-1.7	-1.75	-1.8	-1.85	-1.9	-1.95	-2	$\boldsymbol{\lambda}$
33.835	33.935	34.035	34.135	34.235	34.335	34.435	34.535	34.635	34.735	$\boldsymbol{Z}$
0	0	0	0	0	0	0	0	0	0	$\boldsymbol{y_1^*}$
1	1	1	1	1	1	1	1	1	1	$\boldsymbol{y_2^*}$
0	0	0	0	0	0	0	0	0	0	$\boldsymbol{y_3^*}$
1	1	1	1	1	1	1	1	1	1	$\boldsymbol{y_4^*}$
0	0	0	0	0	0	0	0	0	0	$\boldsymbol{y_5^*}$
0	0	0	0	0	0	0	0	0	0	$\boldsymbol{x_1^*}$
7	7	7	7	7	7	7	7	7	7	$\boldsymbol{x_2^*}$
0	0	0	0	0	0	0	0	0	0	$\boldsymbol{x_3^*}$
6	6	6	6	6	6	6	6	6	6	$\boldsymbol{x_4^*}$
0	0	0	0	0	0	0	0	0	0	$\boldsymbol{x_5^*}$
2	2	2	2	2	2	2	2	2	2	$\boldsymbol{K}$

Table 1: Information on the objective function and variables for different values of λ (Continued).

-1.05	-1.1	-1.15	-1.2	-1.25	-1.3	-1.35	-1.4	-1.45	-1.5	$\boldsymbol{\lambda}$
32.877	32.935	33.035	33.135	33.235	33.335	33.435	33.535	33.635	33.735	$\boldsymbol{Z}$
0	0	0	0	0	0	0	0	0	0	$\boldsymbol{y_1^*}$
1	1	1	1	1	1	1	1	1	1	$\boldsymbol{y_2^*}$
0	0	0	0	0	0	0	0	0	0	$\boldsymbol{y_3^*}$
0	1	1	1	1	1	1	1	1	1	$\boldsymbol{y_4^*}$
0	0	0	0	0	0	0	0	0	0	$\boldsymbol{y_5^*}$
0	0	0	0	0	0	0	0	0	0	$\boldsymbol{x_1^*}$
11	7	7	7	7	7	7	7	7	7	$\boldsymbol{x_2^*}$
0	0	0	0	0	0	0	0	0	0	$\boldsymbol{x_3^*}$
0	6	6	6	6	6	6	6	6	6	$\boldsymbol{x_4^*}$
0	0	0	0	0	0	0	0	0	0	$\boldsymbol{x_5^*}$
1	2	2	2	2	2	2	2	2	2	$\boldsymbol{K}$

Table 1: Information on the objective function and variables for different values of λ (Continued).

-0.55	-0.6	-0.65	-0.7	-0.75	-0.8	-0.85	-0.9	-0.95	-1	$\boldsymbol{\lambda}$
32.377	32.427	32.477	32.527	32.577	32.627	32.677	32.727	32.777	32.827	$\boldsymbol{Z}$
0	0	0	0	0	0	0	0	0	0	$\boldsymbol{y_1^*}$
1	1	1	1	1	1	1	1	1	1	$\boldsymbol{y_2^*}$
0	0	0	0	0	0	0	0	0	0	$\boldsymbol{y_3^*}$
0	0	0	0	0	0	0	0	0	0	$\boldsymbol{y_4^*}$
0	0	0	0	0	0	0	0	0	0	$\boldsymbol{y_5^*}$
0	0	0	0	0	0	0	0	0	0	$\boldsymbol{x_1^*}$
11	11	11	11	11	11	11	11	11	11	$\boldsymbol{x_2^*}$
0	0	0	0	0	0	0	0	0	0	$\boldsymbol{x_3^*}$
0	0	0	0	0	0	0	0	0	0	$\boldsymbol{x_4^*}$
0	0	0	0	0	0	0	0	0	0	$\boldsymbol{x_5^*}$
1	1	1	1	1	1	1	1	1	1	$\boldsymbol{K}$

Table 1: Information on the objective function and variables for different values of λ (Continued).

-0.05	-0.1	-0.15	-0.2	-0.25	-0.3	-0.35	-0.4	-0.45	-0.5	$\boldsymbol{\lambda}$
31.877	31.927	31.977	32.027	32.077	32.127	32.177	32.227	32.277	32.327	$\boldsymbol{Z}$
0	0	0	0	0	0	0	0	0	0	y_1^*
1	1	1	1	1	1	1	1	1	1	y_2^*
0	0	0	0	0	0	0	0	0	0	y_3^*
0	0	0	0	0	0	0	0	0	0	y_4^*
0	0	0	0	0	0	0	0	0	0	y_5^*
0	0	0	0	0	0	0	0	0	0	x_1^*
11	11	11	11	11	11	11	11	11	11	x_2^*
0	0	0	0	0	0	0	0	0	0	x_3^*
0	0	0	0	0	0	0	0	0	0	x_4^*
0	0	0	0	0	0	0	0	0	0	x_5^*
1	1	1	1	1	1	1	1	1	1	$\boldsymbol{K}$

Table 1: Information on the objective function and variables for different values of λ (Continued).

0.45	0.4	0.35	0.3	0.25	0.2	0.15	0.1	0.05	0	$\boldsymbol{\lambda}$
31.377	31.427	31.477	31.527	31.577	31.627	31.677	31.727	31.777	31.827	$\boldsymbol{Z}$
0	0	0	0	0	0	0	0	0	0	y_1^*
1	1	1	1	1	1	1	1	1	1	y_2^*
0	0	0	0	0	0	0	0	0	0	y_3^*
0	0	0	0	0	0	0	0	0	0	y_4^*
0	0	0	0	0	0	0	0	0	0	y_5^*
0	0	0	0	0	0	0	0	0	0	x_1^*
11	11	11	11	11	11	11	11	11	11	x_2^*
0	0	0	0	0	0	0	0	0	0	x_3^*
0	0	0	0	0	0	0	0	0	0	x_4^*
0	0	0	0	0	0	0	0	0	0	x_5^*
1	1	1	1	1	1	1	1	1	1	$\boldsymbol{K}$

Table 1: Information on the objective function and variables for different values of λ (Continued).

0.95	0.9	0.85	0.8	0.75	0.7	0.65	0.6	0.55	0.5	$\boldsymbol{\lambda}$
30.877	30.927	30.977	31.027	31.077	31.127	31.177	31.227	31.277	31.327	$\boldsymbol{Z}$
0	0	0	0	0	0	0	0	0	0	y_1^*
1	1	1	1	1	1	1	1	1	1	y_2^*
0	0	0	0	0	0	0	0	0	0	y_3^*
0	0	0	0	0	0	0	0	0	0	y_4^*
0	0	0	0	0	0	0	0	0	0	y_5^*
0	0	0	0	0	0	0	0	0	0	x_1^*
11	11	11	11	11	11	11	11	11	11	x_2^*
0	0	0	0	0	0	0	0	0	0	x_3^*
0	0	0	0	0	0	0	0	0	0	x_4^*
0	0	0	0	0	0	0	0	0	0	x_5^*
1	1	1	1	1	1	1	1	1	1	$\boldsymbol{K}$

Table 1: Information on the objective function and variables for different values of λ (Continued).

1.45	1.4	1.35	1.3	1.25	1.2	1.15	1.1	1.05	1	$\boldsymbol{\lambda}$
30.377	30.427	30.477	30.527	30.577	30.627	30.677	30.727	30.777	30.827	$\boldsymbol{Z}$
0	0	0	0	0	0	0	0	0	0	$\boldsymbol{y_1^*}$
1	1	1	1	1	1	1	1	1	1	$\boldsymbol{y_2^*}$
0	0	0	0	0	0	0	0	0	0	$\boldsymbol{y_3^*}$
0	0	0	0	0	0	0	0	0	0	$\boldsymbol{y_4^*}$
0	0	0	0	0	0	0	0	0	0	$\boldsymbol{y_5^*}$
0	0	0	0	0	0	0	0	0	0	$\boldsymbol{x_1^*}$
11	11	11	11	11	11	11	11	11	11	$\boldsymbol{x_2^*}$
0	0	0	0	0	0	0	0	0	0	$\boldsymbol{x_3^*}$
0	0	0	0	0	0	0	0	0	0	$\boldsymbol{x_4^*}$
0	0	0	0	0	0	0	0	0	0	$\boldsymbol{x_5^*}$
1	1	1	1	1	1	1	1	1	1	$\boldsymbol{K}$

Table 1: Information on the objective function and variables for different values of λ (Continued).

2	1.95	1.9	1.85	1.8	1.75	1.7	1.65	1.6	1.55	1.5	$\boldsymbol{\lambda}$
29.827	29.877	29.927	29.977	30.027	30.077	30.127	30.177	30.227	30.277	30.327	$\boldsymbol{Z}$
0	0	0	0	0	0	0	0	0	0	0	$\boldsymbol{y_1^*}$
1	1	1	1	1	1	1	1	1	1	1	$\boldsymbol{y_2^*}$
0	0	0	0	0	0	0	0	0	0	0	$\boldsymbol{y_3^*}$
0	0	0	0	0	0	0	0	0	0	0	$\boldsymbol{y_4^*}$
0	0	0	0	0	0	0	0	0	0	0	$\boldsymbol{y_5^*}$
0	0	0	0	0	0	0	0	0	0	0	$\boldsymbol{x_1^*}$
11	11	11	11	11	11	11	11	11	11	11	$\boldsymbol{x_2^*}$
0	0	0	0	0	0	0	0	0	0	0	$\boldsymbol{x_3^*}$
0	0	0	0	0	0	0	0	0	0	0	$\boldsymbol{x_4^*}$
0	0	0	0	0	0	0	0	0	0	0	$\boldsymbol{x_5^*}$
1	1	1	1	1	1	1	1	1	1	1	$\boldsymbol{K}$
