# Supplementary material for: Constrained portfolio optimization with discrete variables: An algorithmic method based on dynamic programming
Source: PLoS One. 2022 Jul 28;17(7):e0271811. doi: 10.1371/journal.pone.0271811 (PMC9333297; doi:10.1371/journal.pone.0271811)
Supplement: S4 Appendix — (PDF) [file pone.0271811.s004.pdf]

```
function
[Sol,R_satr]=Dynamic_prog(Lambda_star,dimx,Pi,Ri,lb,ub,N,vlb,vub,flag)

Interval=struct('a',vlb,'b',vub);
n_r=numel(Interval.a);

data=struct('PN',[],'RN',[],'PNm',[],'RNm',[],'PT',[],'RT',[]);
data_f=struct('R_star_N',0,'PT',0);

for n=N:-1:1
    n_c=ub(n)-lb(n)+1;
    xval=lb(n):ub(n);
    current_Table=cell_table(n_r,1+n_c+2,data,data_f,Interval);


    if n==N

        wN=xval*Pi(n);
        index_x=find_interval(wN,Interval);
        R_mat=zeros(n_r,n_c);
        for jj=1:numel(xval)

            ii=index_x(jj);
            if ii>0
                xj=xval(jj);
                R_mat(ii,jj)=xj*Ri(n)-Lambda_star;

data0=struct('PN',wN(jj),'RN',R_mat(ii,jj),'PNm',[],'RNm',[],'PT',[],'RT
',[]);
                current_Table{ii,jj+1}=data0;
            end
        end
        [mmax,indexm]=max(R_mat,[],2);
        for ii=1:n_r
            X=nan(dimx,1);
            if mmax(ii)>0

data_f0=struct('R_star_N',mmax(ii),'PT',current_Table{ii,indexm(ii)+1}.P
N);
                X(n)=xval(indexm(ii));
            else
               data_f0=data_f;
            end
```

```
            current_Table{ii,end-1}=data_f0;
             current_Table{ii,end}=X;
        end
      if strcmpi(flag,'print')
          Print_Table(current_Table,n,xval)
      end

        current_Table00=current_Table;
    else
        wj=xval*Pi(n);
        index_x=find_interval(wj,Interval);
        R_mat=zeros(n_r,n_c);
        P_mat=zeros(n_r,n_c);
        R_T_mat=zeros(n_r,n_c);
        P_T_mat=zeros(n_r,n_c);
        PNm1=zeros(n_r,n_c);
        RNm1=zeros(n_r,n_c);

        for jj=1:numel(xval)


            xj=xval(jj);

            ii=index_x(jj);
            if ii>0
                R_mat(ii,jj)=xj*Ri(n)-Lambda_star;
                P_mat(ii,jj)=wj(jj);
                ZijNm1=Interval.b(ii)-wj(jj);
                index_z=find_interval(ZijNm1,Interval);
                if ZijNm1>current_Table00{index_z,end-1}.PT
                    RNm1(ii,jj)=current_Table00{index_z,end-1}.R_star_N;
                    PNm1(ii,jj)=current_Table00{index_z,end-1}.PT;
                else
                    if index_z-1>=1
                        PNm1(ii,jj)=current_Table00{index_z-1,end-1}.PT;
                        RNm1(ii,jj)=current_Table00{index_z-1,end-
1}.R_star_N;
                    end
                end
                R_T_mat(ii,jj)=R_mat(ii,jj)+RNm1(ii,jj);
                P_T_mat(ii,jj)=wj(jj)+PNm1(ii,jj);


data0=struct('PN',wj(jj),'RN',R_mat(ii,jj),'PNm',PNm1(ii,jj),'RNm',RNm1(
ii,jj),'PT',P_T_mat(ii,jj),'RT',R_T_mat(ii,jj));
                current_Table{ii,jj+1}=data0;
            end
```

```
            for i1=1:n_r
               if i1~=ii

                   qljNm1=Interval.b(i1)-wj(jj);


                   if qljNm1>0
                        index_q=find_interval(qljNm1,Interval);

                        if (current_Table00{index_q,end-1}.PT) &
qljNm1>current_Table00{index_q,end-1}.PT
%------------------------------------------------------------------
                  RNm1(i1,jj)=current_Table00{index_q,end-1}.R_star_N;
                  PNm1(i1,jj)=current_Table00{index_q,end-1}.PT;
%----------------------------------------------------------------------
                       else
                 if index_q-1>=1 & (current_Table00{index_q-1,end-1}.PT)
%----------------------------------------------------------------------

                 RNm1(i1,jj)=current_Table00{index_q-1,end-1}.R_star_N;
                 PNm1(i1,jj)=current_Table00{index_q-1,end-1}.PT;
%---------------------------------------------------------------------
                            else
                            if ~(current_Table00{index_q,end-1}.PT)
                                P_mat(i1,jj)=0;
                                R_mat(i1,jj)=0;
                         RNm1(i1,jj)=current_Table00{i1,end-1}.R_star_N;
                                PNm1(i1,jj)=current_Table00{i1,end-1}.PT;

                            elseif  (current_Table00{i1,end-1}.PT)
                                P_mat(i1,jj)=0;
                                R_mat(i1,jj)=0;
                         RNm1(i1,jj)=current_Table00{i1,end-1}.R_star_N;
                                PNm1(i1,jj)=current_Table00{i1,end-1}.PT;

                            end
                        end

                       end
                       Kes=PNm1(i1,jj)+wj(jj);


                       if  Kes>=Interval.a(i1) && Kes<Interval.b(i1)

                          R_mat(i1,jj)=xj*Ri(n)-Lambda_star;
                          P_mat(i1,jj)=wj(jj);
```

```
                    else
                       R_mat(i1,jj)=0;
                       P_mat(i1,jj)=0;

                    end

                else

                    P_mat(i1,jj)=0;
                    R_mat(i1,jj)=0;
                    RNm1(i1,jj)=current_Table00{i1,end-1}.R_star_N;
                    PNm1(i1,jj)=current_Table00{i1,end-1}.PT;

                end
                  P_T_mat(i1,jj)=P_mat(i1,jj)+PNm1(i1,jj);
                  R_T_mat(i1,jj)=R_mat(i1,jj)+RNm1(i1,jj);

            end

data0=struct('PN',P_mat(i1,jj),'RN',R_mat(i1,jj),'PNm',PNm1(i1,jj),'RNm'
,RNm1(i1,jj),'PT',P_T_mat(i1,jj),'RT',R_T_mat(i1,jj));
              current_Table{i1,jj+1}=data0;
           end



        end

        [mmax,indexm]=max(R_T_mat,[],2);
        for iii=1:n_r
             X=nan(dimx,1);
            if mmax(iii)>0

data_f0=struct('R_star_N',mmax(iii),'PT',current_Table{iii,indexm(iii)+1
}.PT);
                if R_mat(iii,indexm(iii))>0
                    X(n)=xval(indexm(iii));
                end

                case_data=RNm1(iii,indexm(iii));
                if case_data>0
                    index_ii=find_interval2(current_Table00,case_data);
                    X2=current_Table00{index_ii,end};
                    other_ind=find(X2);
                    if ~isempty(other_ind)
                        other_ind(other_ind==n)=[];
                        X(other_ind)=X2(other_ind);
```

```
                    end
                end


            else
                data_f0=data_f;
            end
            current_Table{iii,end-1}=data_f0;
            current_Table{iii,end}=X;
        end
        if strcmpi(flag,'print')
            Print_Table(current_Table,n,xval)
        end
        current_Table00=current_Table;
    end

end
RS=zeros(n_r,1);
for ii=1:n_r
    RS(ii)=current_Table{ii,end-1}.R_star_N;
end

[R_satr,indmax]=max(RS);

Sol=current_Table{indmax,end};
Sol(isnan(Sol))=0;
```

:cell_table -

```
function z=cell_table(n_r,n_c,data,data_f,Interval)

z=cell(n_r,n_c);
for ii=1:n_r
    I.a=Interval.a(ii);
    I.b=Interval.b(ii);
    z{ii,1}=I;
    for jj=2:n_c-2
        z{ii,jj}=data;
    end
    z{ii,n_c-1}=data_f;

end
```

:find_interval -

```
   function z=find_interval(wN,Interval)

L=Interval.a;
U=Interval.b;
z=-ones(size(wN)); % if Interval doesnt include the wN
for ii=1:numel(wN)
    op=find(wN(ii)>=L & wN(ii)<U);
    if ~isempty(op)
        z(ii)=op;
    end

end
```

::find_interval2 -

```
function z=find_interval2(T,data)

w=zeros(size(T,1),1);
for ii=1:size(T,1)
    w(ii)=T{ii,end-1}.R_star_N;
end

z=find(abs(w-data)<10^-5);
```

Print_Table –

```
function Print_Table(T,num,xval)


name=strcat('Table-',int2str(num),'.txt');

id=fopen(name,'a');

[r,c]=size(T);
for ii=1:r
    ST=[];
    for jj=1:c
        ss=T{ii,jj};
       if jj==1
           st0=sprintf('[%0.2f , %0.2f)  :',ss.a,ss.b);
           st0x=blanks(numel(st0)+3);

       elseif jj>1 && jj<=c-2
           fields = fieldnames(ss);
           st=[];
           nf1=numel(fields);% +1 because of x value on top of each
interval

           for k=1:nf1
               val=getfield(ss,fields{k});
               if isempty(val)
                   s_val='-';
               else
                   s_val= num2str(val);
               end
               st=strvcat(st,strcat(fields{k},'=',s_val));
           end

           st=strvcat(num2str(xval(jj-1)),st);
           ST= [ST repmat(blanks(3),nf1+1,1) st];

       elseif jj==c-1
            ST=[ST repmat('     ',nf1+1,1)];
            fields = fieldnames(ss);
            st=[];
            nf2=numel(fields);
            for k=1:nf2
               val=getfield(ss,fields{k});
```

```
                if isempty(val)
                    s_val='-';
                else
                    s_val= num2str(val);
                end
                st=strvcat(st,strcat(fields{k},'=',s_val));
            end
            st2=repmat([blanks(size(st,2)-1) ','],nf1+1,1);
            st2(1:size(st,1),:)=st;
            ST=[ST st2 repmat('    ',max([nf1+1,nf2]),1)];

        else

            ind=find(~isnan(ss))  ;
            s=[];
            sec=[1 numel(ind)];
            if numel(ind)>50
                sec=1:numel(ind)/50:numel(ind);
                if sec(end)~=numel(ind)
                    sec(end)=numel(ind);
                end
            end
            sp=[];
            for p=1:numel(sec)-1
                s=[];
                for k=sec(p):sec(p+1)
                    s=strcat(s,'(index "',int2str(ind(k)),'"
:',num2str(ss(ind(k))),') ,');
                end
                sp=strvcat(sp,s);
            end

        end
    end
    st02=repmat([blanks(numel(st0)-1) ' '],nf1+1,1);
    st02(floor(nf1/2),:)=st0;

     sp2=repmat([blanks(numel(sp)-1) ' '],nf1+1,1);
    sp2(1:size(sp,1),:)=sp;
        S_=[st02 ST sp2]

        for l=1:size(S_,1)
            fwrite(id ,S_(l,:));
            fprintf(id,'\n');
        end


fprintf(id,'\n_________________________________________________________
```

```
________________________________________________________________________
________________________________________________________________________
______________________________________\n');
       S_=[];
end

fclose(id);
disp(' print was done successfully')

fprintf('\n_____________________________________________________________
________________________________________________________________________
________________________________________________________________________
___________________________________\n');
```

```
clc
clear
close all
format short

name='Initial Data.xlsx';
sheet='Data';

%%%%%%%%%%%%%%%%%%%%%%%%%%%%%%%%%%%%%%%%%%%%%%%%%%%%%%

A=xlsread(name,sheet);
Lambda_star=A(1,1);
dimx=A(1,2);

Pi=A(:,4);
Ri=A(:,5);
lb=A(:,6);
ub=A(:,7);
B=A(1,8);
L=A(1,9);

N=numel(Pi);
vL=0:L:B;
vL(end)=B;

vlb=vL(1:end-1);
vub=vL(2:end);

[Solution_a,Ra]=Dynamic_prog(Lambda_star,dimx,Pi,Ri,lb,ub,N,vlb,vub,'pri
nt');
disp(Ra)

ss=sparse(Solution_a)
disp(L)
```
