# Supplementary material for: Constrained portfolio optimization with discrete variables: An algorithmic method based on dynamic programming
Source: PLoS One. 2022 Jul 28;17(7):e0271811. doi: 10.1371/journal.pone.0271811 (PMC9333297; doi:10.1371/journal.pone.0271811)
Supplement: S5 Appendix — (PDF) [file pone.0271811.s005.pdf]

Table 1: The output of the solution for the fifth environmental share.

```
S_ =

                      5        6        7        8        9        10        R_star_N=0
                      PN=-     PN=-     PN=-     PN=-     PN=-     PN=-      PT=0
[0.00 , 65.00)   :    RN=-     RN=-     RN=-     RN=-     RN=-     RN=-                ,
                      PNm=-    PNm=-    PNm=-    PNm=-    PNm=-    PNm=-               ,
                      RNm=-    RNm=-    RNm=-    RNm=-    RNm=-    RNm=-               ,
                      PT=-     PT=-     PT=-     PT=-     PT=-     PT=-                ,
                      RT=-     RT=-     RT=-     RT=-     RT=-     RT=-                ,
```

```
S_ =

                      5        6        7        8        9        10        R_star_N=0
                      PN=-     PN=-     PN=-     PN=-     PN=-     PN=-      PT=0
[65.00 , 130.00)  :   RN=-     RN=-     RN=-     RN=-     RN=-     RN=-                ,
                      PNm=-    PNm=-    PNm=-    PNm=-    PNm=-    PNm=-               ,
                      RNm=-    RNm=-    RNm=-    RNm=-    RNm=-    RNm=-               ,
                      PT=-     PT=-     PT=-     PT=-     PT=-     PT=-                ,
                      RT=-     RT=-     RT=-     RT=-     RT=-     RT=-                ,
```

```
S_ =

                      5        6        7        8        9        10        R_star_N=0
                      PN=-     PN=-     PN=-     PN=-     PN=-     PN=-      PT=0
[130.00 , 195.00)  :  RN=-     RN=-     RN=-     RN=-     RN=-     RN=-                ,
                      PNm=-    PNm=-    PNm=-    PNm=-    PNm=-    PNm=-               ,
                      RNm=-    RNm=-    RNm=-    RNm=-    RNm=-    RNm=-               ,
                      PT=-     PT=-     PT=-     PT=-     PT=-     PT=-                ,
                      RT=-     RT=-     RT=-     RT=-     RT=-     RT=-                ,
```

```
S_ =

                      5        6        7        8        9        10        R_star_N=0
                      PN=-     PN=-     PN=-     PN=-     PN=-     PN=-      PT=0
[195.00 , 260.00)  :  RN=-     RN=-     RN=-     RN=-     RN=-     RN=-                ,
                      PNm=-    PNm=-    PNm=-    PNm=-    PNm=-    PNm=-               ,
                      RNm=-    RNm=-    RNm=-    RNm=-    RNm=-    RNm=-               ,
                      PT=-     PT=-     PT=-     PT=-     PT=-     PT=-                ,
                      RT=-     RT=-     RT=-     RT=-     RT=-     RT=-                ,
```

```
S_ =

                     5             6      7      8      9      10      R_star_N=8.4965    (index "5" :5) ,
                     PN=287.5965   PN=-   PN=-   PN=-   PN=-   PN=-    PT=287.5965
[260.00 , 325.00)  : RN=8.4965     RN=-   RN=-   RN=-   RN=-   RN=-                ,
                     PNm=-         PNm=-  PNm=-  PNm=-  PNm=-  PNm=-               ,
                     RNm=-         RNm=-  RNm=-  RNm=-  RNm=-  RNm=-               ,
                     PT=-          PT=-   PT=-   PT=-   PT=-   PT=-                ,
                     RT=-          RT=-   RT=-   RT=-   RT=-   RT=-                ,
```

```
S_ =

                     5       6             7      8      9      10      R_star_N=9.9758    (index "5" :6) ,
                     PN=-    PN=345.1158   PN=-   PN=-   PN=-   PN=-    PT=345.1158
[325.00 , 390.00)  : RN=-    RN=9.9758     RN=-   RN=-   RN=-   RN=-                ,
                     PNm=-   PNm=-         PNm=-  PNm=-  PNm=-  PNm=-               ,
                     RNm=-   RNm=-         RNm=-  RNm=-  RNm=-  RNm=-               ,
                     PT=-    PT=-          PT=-   PT=-   PT=-   PT=-                ,
                     RT=-    RT=-          RT=-   RT=-   RT=-   RT=-                ,
```

```
S_ =

                         5      6      7           8      9      10      R_star_N=11.4551    (index "5" :7) ,
                         PN=-   PN=-   PN=402.6351 PN=-   PN=-   PN=-    PT=402.6351
[390.00 , 455.00)  :     RN=-   RN=-   RN=11.4551  RN=-   RN=-   RN=-                ,
                         PNm=-  PNm=-  PNm=-       PNm=-  PNm=-  PNm=-               ,
                         RNm=-  RNm=-  RNm=-       RNm=-  RNm=-  RNm=-               ,
                         PT=-   PT=-   PT=-        PT=-   PT=-   PT=-                ,
                         RT=-   RT=-   RT=-        RT=-   RT=-   RT=-                ,

S_ =

                         5      6      7      8            9            10      R_star_N=14.4137    (index "5" :9) ,
                         PN=-   PN=-   PN=-   PN=460.1544  PN=517.6737  PN=-    PT=517.6737
[455.00 , 520.00)  :     RN=-   RN=-   RN=-   RN=12.9344   RN=14.4137   RN=-                ,
                         PNm=-  PNm=-  PNm=-  PNm=-        PNm=-        PNm=-               ,
                         RNm=-  RNm=-  RNm=-  RNm=-        RNm=-        RNm=-               ,
                         PT=-   PT=-   PT=-   PT=-         PT=-         PT=-                ,
                         RT=-   RT=-   RT=-   RT=-         RT=-         RT=-                ,

S_ =

                         5      6      7      8      9      10           R_star_N=15.893    (index "5" :10) ,
                         PN=-   PN=-   PN=-   PN=-   PN=-   PN=575.193   PT=575.193
[520.00 , 585.00)  :     RN=-   RN=-   RN=-   RN=-   RN=-   RN=15.893               ,
                         PNm=-  PNm=-  PNm=-  PNm=-  PNm=-  PNm=-                   ,
                         RNm=-  RNm=-  RNm=-  RNm=-  RNm=-  RNm=-                   ,
                         PT=-   PT=-   PT=-   PT=-   PT=-   PT=-                    ,
                         RT=-   RT=-   RT=-   RT=-   RT=-   RT=-                    ,

S_ =

                         5      6      7      8      9      10      R_star_N=0
                         PN=-   PN=-   PN=-   PN=-   PN=-   PN=-    PT=0
[585.00 , 650.00)  :     RN=-   RN=-   RN=-   RN=-   RN=-   RN=-              ,
                         PNm=-  PNm=-  PNm=-  PNm=-  PNm=-  PNm=-             ,
                         RNm=-  RNm=-  RNm=-  RNm=-  RNm=-  RNm=-             ,
                         PT=-   PT=-   PT=-   PT=-   PT=-   PT=-              ,
                         RT=-   RT=-   RT=-   RT=-   RT=-   RT=-              ,
```

Table 2: The output of the solution for the fourth environmental share.

```
S_ =

                          5        6        7        8        9        10       11         R_star_N=0
                          PN=0     PN=0     PN=0     PN=0     PN=0     PN=0     PN=0       PT=0
[0.00 , 65.00)  :         RN=0     RN=0     RN=0     RN=0     RN=0     RN=0     RN=0                 ,
                          PNm=0    PNm=0    PNm=0    PNm=0    PNm=0    PNm=0    PNm=0                ,
                          RNm=0    RNm=0    RNm=0    RNm=0    RNm=0    RNm=0    RNm=0                ,
                          PT=0     PT=0     PT=0     PT=0     PT=0     PT=0     PT=0                 ,
                          RT=0     RT=0     RT=0     RT=0     RT=0     RT=0     RT=0                 ,
```

```
S_ =

                          5        6        7        8        9        10       11         R_star_N=0
                          PN=0     PN=0     PN=0     PN=0     PN=0     PN=0     PN=0       PT=0
[65.00 , 130.00)  :       RN=0     RN=0     RN=0     RN=0     RN=0     RN=0     RN=0                 ,
                          PNm=0    PNm=0    PNm=0    PNm=0    PNm=0    PNm=0    PNm=0                ,
                          RNm=0    RNm=0    RNm=0    RNm=0    RNm=0    RNm=0    RNm=0                ,
                          PT=0     PT=0     PT=0     PT=0     PT=0     PT=0     PT=0                 ,
                          RT=0     RT=0     RT=0     RT=0     RT=0     RT=0     RT=0                 ,
```

```
S_ =

                          5        6        7        8        9        10       11         R_star_N=0
                          PN=0     PN=0     PN=0     PN=0     PN=0     PN=0     PN=0       PT=0
[130.00 , 195.00)  :      RN=0     RN=0     RN=0     RN=0     RN=0     RN=0     RN=0                 ,
                          PNm=0    PNm=0    PNm=0    PNm=0    PNm=0    PNm=0    PNm=0                ,
                          RNm=0    RNm=0    RNm=0    RNm=0    RNm=0    RNm=0    RNm=0                ,
                          PT=0     PT=0     PT=0     PT=0     PT=0     PT=0     PT=0                 ,
                          RT=0     RT=0     RT=0     RT=0     RT=0     RT=0     RT=0                 ,
```

```
S_ =

                       5              6              7              8              9              R_star_N=9.834    (index "4" :5) ,
                       PN=0           PN=0           PN=0           PN=0           PN=0           PT=242.807
[195.00 , 260.00)  :   RN=0           RN=0           RN=0           RN=0           RN=0                            ,
                       PNm=242.807    PNm=242.807    PNm=242.807    PNm=242.807    PNm=242.807                     ,
                       RNm=9.834      RNm=9.834      RNm=9.834      RNm=9.834      RNm=9.834                       ,
                       PT=242.807     PT=242.807     PT=242.807     PT=242.807     PT=242.807                      ,
                       RT=9.834       RT=9.834       RT=9.834       RT=9.834       RT=9.834                        ,
```

```
S_ =

                       5               6               7               8               9               R_star_N=11.5808    (index "4" :6) ,
                       PN=0            PN=0            PN=0            PN=0            PN=0            PT=291.3684
[260.00 , 325.00)  :   RN=0            RN=0            RN=0            RN=0            RN=0                              ,
                       PNm=291.3684    PNm=291.3684    PNm=291.3684    PNm=291.3684    PNm=291.3684                      ,
                       RNm=11.5808     RNm=11.5808     RNm=11.5808     RNm=11.5808     RNm=11.5808                       ,
                       PT=291.3684     PT=291.3684     PT=291.3684     PT=291.3684     PT=291.3684                       ,
                       RT=11.5808      RT=11.5808      RT=11.5808      RT=11.5808      RT=11.5808                        ,
```

S_ =

	5	6	7	8	9	R_star_N=17.8975	(index "3" :5) ,
	PN=362.8535	PN=0	PN=0	PN=0	PN=0	PT=362.8535	
[325.00 , 390.00) :	RN=17.8975	RN=0	RN=0	RN=0	RN=0	,	
	PNm=0	PNm=388.4912	PNm=388.4912	PNm=388.4912	PNm=388.4912	,	
	RNm=0	RNm=15.0744	RNm=15.0744	RNm=15.0744	RNm=15.0744	,	
	PT=362.8535	PT=388.4912	PT=388.4912	PT=388.4912	PT=388.4912	,	
	RT=17.8975	RT=15.0744	RT=15.0744	RT=15.0744	RT=15.0744	,	

S_ =

	5	6	7	8	9	R_star_N=21.257	(index "3" :6) ,
	PN=0	PN=435.4242	PN=0	PN=0	PN=0	PT=435.4242	
[390.00 , 455.00) :	RN=0	RN=21.257	RN=0	RN=0	RN=0	,	
	PNm=437.0526	PNm=0	PNm=437.0526	PNm=437.0526	PNm=437.0526	,	
	RNm=16.8212	RNm=0	RNm=16.8212	RNm=16.8212	RNm=16.8212	,	
	PT=437.0526	PT=435.4242	PT=437.0526	PT=437.0526	PT=437.0526	,	
	RT=16.8212	RT=21.257	RT=16.8212	RT=16.8212	RT=16.8212	,	

S_ =

	5	6	7	8	9	R_star_N=24.6165	(index "3" :7) ,
	PN=0	PN=0	PN=507.9949	PN=0	PN=0	PT=507.9949	
[455.00 , 520.00) :	RN=0	RN=0	RN=24.6165	RN=0	RN=0	,	
	PNm=485.614	PNm=485.614	PNm=0	PNm=485.614	PNm=485.614	,	
	RNm=18.568	RNm=18.568	RNm=0	RNm=18.568	RNm=18.568	,	
	PT=485.614	PT=485.614	PT=507.9949	PT=485.614	PT=485.614	,	
	RT=18.568	RT=18.568	RT=24.6165	RT=18.568	RT=18.568	,	

S_ =

	5	6	7	8	9	R_star_N=27.976	(index "3" :8) ,
	PN=0	PN=0	PN=0	PN=580.5656	PN=0	PT=580.5656	
[520.00 , 585.00) :	RN=0	RN=0	RN=0	RN=27.976	RN=0	,	
	PNm=534.1754	PNm=534.1754	PNm=534.1754	PNm=0	PNm=534.1754	,	
	RNm=20.3148	RNm=20.3148	RNm=20.3148	RNm=0	RNm=20.3148	,	
	PT=534.1754	PT=534.1754	PT=534.1754	PT=580.5656	PT=534.1754	,	
	RT=20.3148	RT=20.3148	RT=20.3148	RT=27.976	RT=20.3148	,	

S_ =

	5	6	7	8	9	10	11	R_star_N=21.8241	(index "4" :7) ,(index "5" :5) ,
	PN=242.807	PN=291.3684	PN=339.9298	PN=0	PN=0	PN=0	PN=0	PT=627.5263	
[585.00 , 650.00) :	RN=9.834	RN=11.5808	RN=13.3276	RN=0	RN=0	RN=0	RN=0	,	
	PNm=402.6351	PNm=345.1158	PNm=287.5965	PNm=0	PNm=0	PNm=0	PNm=0	,	
	RNm=11.4551	RNm=9.9758	RNm=8.4965	RNm=0	RNm=0	RNm=0	RNm=0	,	
	PT=645.4421	PT=636.4842	PT=627.5263	PT=0	PT=0	PT=0	PT=0	,	
	RT=21.2891	RT=21.5566	RT=21.8241	RT=0	RT=0	RT=0	RT=0	,	

Table 3: The output of the solution for the third environmental share.

```
S_ =

                    5        6        7        8        9         R_star_N=0
                    PN=0     PN=0     PN=0     PN=0     PN=0      PT=0
[0.00 , 65.00)  :   RN=0     RN=0     RN=0     RN=0     RN=0              ,
                    PNm=0    PNm=0    PNm=0    PNm=0    PNm=0             ,
                    RNm=0    RNm=0    RNm=0    RNm=0    RNm=0             ,
                    PT=0     PT=0     PT=0     PT=0     PT=0              ,
                    RT=0     RT=0     RT=0     RT=0     RT=0              ,

S_ =

                     5        6        7        8        9         R_star_N=0
                     PN=0     PN=0     PN=0     PN=0     PN=0      PT=0
[65.00 , 130.00)  :  RN=0     RN=0     RN=0     RN=0     RN=0              ,
                     PNm=0    PNm=0    PNm=0    PNm=0    PNm=0             ,
                     RNm=0    RNm=0    RNm=0    RNm=0    RNm=0             ,
                     PT=0     PT=0     PT=0     PT=0     PT=0              ,
                     RT=0     RT=0     RT=0     RT=0     RT=0              ,

S_ =

                      5        6        7        8        9         R_star_N=0
                      PN=0     PN=0     PN=0     PN=0     PN=0      PT=0
[130.00 , 195.00)  :  RN=0     RN=0     RN=0     RN=0     RN=0              ,
                      PNm=0    PNm=0    PNm=0    PNm=0    PNm=0             ,
                      RNm=0    RNm=0    RNm=0    RNm=0    RNm=0             ,
                      PT=0     PT=0     PT=0     PT=0     PT=0              ,
                      RT=0     RT=0     RT=0     RT=0     RT=0              ,

S_ =

                     5              6              7              8              9              R_star_N=9.834    (index "4" :5) ,
                     PN=0           PN=0           PN=0           PN=0           PN=0           PT=242.807
[195.00 , 260.00)  : RN=0           RN=0           RN=0           RN=0           RN=0                     ,
                     PNm=242.807    PNm=242.807    PNm=242.807    PNm=242.807    PNm=242.807              ,
                     RNm=9.834      RNm=9.834      RNm=9.834      RNm=9.834      RNm=9.834                ,
                     PT=242.807     PT=242.807     PT=242.807     PT=242.807     PT=242.807               ,
                     RT=9.834       RT=9.834       RT=9.834       RT=9.834       RT=9.834                 ,

S_ =

                     5              6              7              8              9              R_star_N=11.5808    (index "4" :6) ,
                     PN=0           PN=0           PN=0           PN=0           PN=0           PT=291.3684
[260.00 , 325.00)  : RN=0           RN=0           RN=0           RN=0           RN=0                     ,
                     PNm=291.3684   PNm=291.3684   PNm=291.3684   PNm=291.3684   PNm=291.3684             ,
                     RNm=11.5808    RNm=11.5808    RNm=11.5808    RNm=11.5808    RNm=11.5808              ,
                     PT=291.3684    PT=291.3684    PT=291.3684    PT=291.3684    PT=291.3684              ,
                     RT=11.5808     RT=11.5808     RT=11.5808     RT=11.5808     RT=11.5808               ,

S_ =

                     5              6              7              8              9              R_star_N=17.8975    (index "3" :5) ,
                     PN=362.8535    PN=0           PN=0           PN=0           PN=0           PT=362.8535
[325.00 , 390.00)  : RN=17.8975     RN=0           RN=0           RN=0           RN=0                     ,
                     PNm=0          PNm=388.4912   PNm=388.4912   PNm=388.4912   PNm=388.4912             ,
                     RNm=0          RNm=15.0744    RNm=15.0744    RNm=15.0744    RNm=15.0744              ,
                     PT=362.8535    PT=388.4912    PT=388.4912    PT=388.4912    PT=388.4912              ,
                     RT=17.8975     RT=15.0744     RT=15.0744     RT=15.0744     RT=15.0744               ,
```

S_ =

		5	6	7	8	9	R_star_N=21.257	(index "3" :6) ,
		PN=0	PN=435.4242	PN=0	PN=0	PN=0	PT=435.4242	
[390.00 , 455.00)	:	RN=0	RN=21.257	RN=0	RN=0	RN=0	,	
		PNm=437.0526	PNm=0	PNm=437.0526	PNm=437.0526	PNm=437.0526	,	
		RNm=16.8212	RNm=0	RNm=16.8212	RNm=16.8212	RNm=16.8212	,	
		PT=437.0526	PT=435.4242	PT=437.0526	PT=437.0526	PT=437.0526	,	
		RT=16.8212	RT=21.257	RT=16.8212	RT=16.8212	RT=16.8212	,	

S_ =

		5	6	7	8	9	R_star_N=24.6165	(index "3" :7) ,
		PN=0	PN=0	PN=507.9949	PN=0	PN=0	PT=507.9949	
[455.00 , 520.00)	:	RN=0	RN=0	RN=24.6165	RN=0	RN=0	,	
		PNm=485.614	PNm=485.614	PNm=0	PNm=485.614	PNm=485.614	,	
		RNm=18.568	RNm=18.568	RNm=0	RNm=18.568	RNm=18.568	,	
		PT=485.614	PT=485.614	PT=507.9949	PT=485.614	PT=485.614	,	
		RT=18.568	RT=18.568	RT=24.6165	RT=18.568	RT=18.568	,	

S_ =

		5	6	7	8	9	R_star_N=27.976	(index "3" :8) ,
		PN=0	PN=0	PN=0	PN=580.5656	PN=0	PT=580.5656	
[520.00 , 585.00)	:	RN=0	RN=0	RN=0	RN=27.976	RN=0	,	
		PNm=534.1754	PNm=534.1754	PNm=534.1754	PNm=0	PNm=534.1754	,	
		RNm=20.3148	RNm=20.3148	RNm=20.3148	RNm=0	RNm=20.3148	,	
		PT=534.1754	PT=534.1754	PT=534.1754	PT=580.5656	PT=534.1754	,	
		RT=20.3148	RT=20.3148	RT=20.3148	RT=27.976	RT=20.3148	,	

S_ =

		5	6	7	8	9	R_star_N=27.7315	(index "3" :5) ,(index "4" :5) ,
		PN=362.8535	PN=0	PN=0	PN=0	PN=0	PT=605.6605	
[585.00 , 650.00)	:	RN=17.8975	RN=0	RN=0	RN=0	RN=0	,	
		PNm=242.807	PNm=627.5263	PNm=627.5263	PNm=627.5263	PNm=627.5263	,	
		RNm=9.834	RNm=21.8241	RNm=21.8241	RNm=21.8241	RNm=21.8241	,	
		PT=605.6605	PT=627.5263	PT=627.5263	PT=627.5263	PT=627.5263	,	
		RT=27.7315	RT=21.8241	RT=21.8241	RT=21.8241	RT=21.8241	,	

Table 4: The output of the solution for the second environmental share.

```
S_ =

                    6        7        8        9        10       11       R_star_N=0
                    PN=0     PN=0     PN=0     PN=0     PN=0     PN=0     PT=0
[0.00 , 65.00)  :   RN=0     RN=0     RN=0     RN=0     RN=0     RN=0              ,
                    PNm=0    PNm=0    PNm=0    PNm=0    PNm=0    PNm=0             ,
                    RNm=0    RNm=0    RNm=0    RNm=0    RNm=0    RNm=0             ,
                    PT=0     PT=0     PT=0     PT=0     PT=0     PT=0              ,
                    RT=0     RT=0     RT=0     RT=0     RT=0     RT=0              ,
```

```
S_ =

                      6        7        8        9        10       11       R_star_N=0
                      PN=0     PN=0     PN=0     PN=0     PN=0     PN=0     PT=0
[65.00 , 130.00)  :   RN=0     RN=0     RN=0     RN=0     RN=0     RN=0              ,
                      PNm=0    PNm=0    PNm=0    PNm=0    PNm=0    PNm=0             ,
                      RNm=0    RNm=0    RNm=0    RNm=0    RNm=0    RNm=0             ,
                      PT=0     PT=0     PT=0     PT=0     PT=0     PT=0              ,
                      RT=0     RT=0     RT=0     RT=0     RT=0     RT=0              ,
```

```
S_ =

                       6        7        8        9        10       11       R_star_N=0
                       PN=0     PN=0     PN=0     PN=0     PN=0     PN=0     PT=0
[130.00 , 195.00)  :   RN=0     RN=0     RN=0     RN=0     RN=0     RN=0              ,
                       PNm=0    PNm=0    PNm=0    PNm=0    PNm=0    PNm=0             ,
                       RNm=0    RNm=0    RNm=0    RNm=0    RNm=0    RNm=0             ,
                       PT=0     PT=0     PT=0     PT=0     PT=0     PT=0              ,
                       RT=0     RT=0     RT=0     RT=0     RT=0     RT=0              ,
```

```
S_ =

                       6            7            8            9            10           11           R_star_N=9.834    (index "4" :5) ,
                       PN=0         PN=0         PN=0         PN=0         PN=0         PN=0         PT=242.807
[195.00 , 260.00)  :   RN=0         RN=0         RN=0         RN=0         RN=0         RN=0                   ,
                       PNm=242.807  PNm=242.807  PNm=242.807  PNm=242.807  PNm=242.807  PNm=242.807            ,
                       RNm=9.834    RNm=9.834    RNm=9.834    RNm=9.834    RNm=9.834    RNm=9.834              ,
                       PT=242.807   PT=242.807   PT=242.807   PT=242.807   PT=242.807   PT=242.807             ,
                       RT=9.834     RT=9.834     RT=9.834     RT=9.834     RT=9.834     RT=9.834               ,
```

```
S_ =

                       6            7             8             9             10            11            R_star_N=18.4604    (index "2" :6) ,
                       PN=305.8248  PN=0          PN=0          PN=0          PN=0          PN=0          PT=305.8248
[260.00 , 325.00)  :   RN=18.4604   RN=0          RN=0          RN=0          RN=0          RN=0                    ,
                       PNm=0        PNm=291.3684  PNm=291.3684  PNm=291.3684  PNm=291.3684  PNm=291.3684            ,
                       RNm=0        RNm=11.5808   RNm=11.5808   RNm=11.5808   RNm=11.5808   RNm=11.5808             ,
                       PT=305.8248  PT=291.3684   PT=291.3684   PT=291.3684   PT=291.3684   PT=291.3684             ,
                       RT=18.4604   RT=11.5808    RT=11.5808    RT=11.5808    RT=11.5808    RT=11.5808              ,
```

S_ =

	6	7	8	9	10	11	R_star_N=21.3538	(index "2" :7) ,
	PN=0	PN=356.7956	PN=0	PN=0	PN=0	PN=0	PT=356.7956	
[325.00 , 390.00) :	RN=0	RN=21.3538	RN=0	RN=0	RN=0	RN=0	,	
	PNm=362.8535	PNm=0	PNm=362.8535	PNm=362.8535	PNm=362.8535	PNm=362.8535	,	
	RNm=17.8975	RNm=0	RNm=17.8975	RNm=17.8975	RNm=17.8975	RNm=17.8975	,	
	PT=362.8535	PT=356.7956	PT=362.8535	PT=362.8535	PT=362.8535	PT=362.8535	,	
	RT=17.8975	RT=21.3538	RT=17.8975	RT=17.8975	RT=17.8975	RT=17.8975	,	

S_ =

	6	7	8	9	10	11	R_star_N=24.2472	(index "2" :8) ,
	PN=0	PN=0	PN=407.7664	PN=0	PN=0	PN=0	PT=407.7664	
[390.00 , 455.00) :	RN=0	RN=0	RN=24.2472	RN=0	RN=0	RN=0	,	
	PNm=435.4242	PNm=435.4242	PNm=0	PNm=435.4242	PNm=435.4242	PNm=435.4242	,	
	RNm=21.257	RNm=21.257	RNm=0	RNm=21.257	RNm=21.257	RNm=21.257	,	
	PT=435.4242	PT=435.4242	PT=407.7664	PT=435.4242	PT=435.4242	PT=435.4242	,	
	RT=21.257	RT=21.257	RT=24.2472	RT=21.257	RT=21.257	RT=21.257	,	

S_ =

	6	7	8	9	10	11	R_star_N=30.034	(index "2" :10) ,
	PN=0	PN=0	PN=0	PN=458.7372	PN=509.708	PN=0	PT=509.708	
[455.00 , 520.00) :	RN=0	RN=0	RN=0	RN=27.1406	RN=30.034	RN=0	,	
	PNm=507.9949	PNm=507.9949	PNm=507.9949	PNm=0	PNm=0	PNm=507.9949	,	
	RNm=24.6165	RNm=24.6165	RNm=24.6165	RNm=0	RNm=0	RNm=24.6165	,	
	PT=507.9949	PT=507.9949	PT=507.9949	PT=458.7372	PT=509.708	PT=507.9949	,	
	RT=24.6165	RT=24.6165	RT=24.6165	RT=27.1406	RT=30.034	RT=24.6165	,	

S_ =

	6	7	8	9	10	11	R_star_N=32.9274	(index "2" :11) ,
	PN=305.8248	PN=0	PN=0	PN=0	PN=0	PN=560.6788	PT=560.6788	
[520.00 , 585.00) :	RN=18.4604	RN=0	RN=0	RN=0	RN=0	RN=32.9274	,	
	PNm=242.807	PNm=580.5656	PNm=580.5656	PNm=580.5656	PNm=580.5656	PNm=0	,	
	RNm=9.834	RNm=27.976	RNm=27.976	RNm=27.976	RNm=27.976	RNm=0	,	
	PT=548.6318	PT=580.5656	PT=580.5656	PT=580.5656	PT=580.5656	PT=560.6788	,	
	RT=28.2944	RT=27.976	RT=27.976	RT=27.976	RT=27.976	RT=32.9274	,	

S_ =

	6	7	8	9	10	11	R_star_N=32.9346	(index "2" :7) ,(index "4" :6)
	PN=305.8248	PN=356.7956	PN=0	PN=0	PN=0	PN=0	PT=648.164	
[585.00 , 650.00) :	RN=18.4604	RN=21.3538	RN=0	RN=0	RN=0	RN=0	,	
	PNm=291.3684	PNm=291.3684	PNm=605.6605	PNm=605.6605	PNm=605.6605	PNm=605.6605	,	
	RNm=11.5808	RNm=11.5808	RNm=27.7315	RNm=27.7315	RNm=27.7315	RNm=27.7315	,	
	PT=597.1932	PT=648.164	PT=605.6605	PT=605.6605	PT=605.6605	PT=605.6605	,	
	RT=30.0412	RT=32.9346	RT=27.7315	RT=27.7315	RT=27.7315	RT=27.7315	,	

Table 5: The output of the solution for the first environmental share.

```
S_ =

                        2        3        4        5        6          R_star_N=0
                        PN=0     PN=0     PN=0     PN=0     PN=0       PT=0
[0.00 , 65.00)   :      RN=0     RN=0     RN=0     RN=0     RN=0                 ,
                        PNm=0    PNm=0    PNm=0    PNm=0    PNm=0                ,
                        RNm=0    RNm=0    RNm=0    RNm=0    RNm=0                ,
                        PT=0     PT=0     PT=0     PT=0     PT=0                 ,
                        RT=0     RT=0     RT=0     RT=0     RT=0                 ,
```

```
S_ =

                         2        3        4        5        6          R_star_N=0
                         PN=0     PN=0     PN=0     PN=0     PN=0       PT=0
[65.00 , 130.00)   :     RN=0     RN=0     RN=0     RN=0     RN=0                 ,
                         PNm=0    PNm=0    PNm=0    PNm=0    PNm=0                ,
                         RNm=0    RNm=0    RNm=0    RNm=0    RNm=0                ,
                         PT=0     PT=0     PT=0     PT=0     PT=0                 ,
                         RT=0     RT=0     RT=0     RT=0     RT=0                 ,
```

```
S_ =

                          2        3        4        5        6          R_star_N=0
                          PN=0     PN=0     PN=0     PN=0     PN=0       PT=0
[130.00 , 195.00)   :     RN=0     RN=0     RN=0     RN=0     RN=0                 ,
                          PNm=0    PNm=0    PNm=0    PNm=0    PNm=0                ,
                          RNm=0    RNm=0    RNm=0    RNm=0    RNm=0                ,
                          PT=0     PT=0     PT=0     PT=0     PT=0                 ,
                          RT=0     RT=0     RT=0     RT=0     RT=0                 ,
```

```
S_ =

                      2             3             4             5             6             R_star_N=9.834    (index "4" :5) ,
                      PN=0          PN=0          PN=0          PN=0          PN=0          PT=242.807
[195.00 , 260.00)  :  RN=0          RN=0          RN=0          RN=0          RN=0                     ,
                      PNm=242.807   PNm=242.807   PNm=242.807   PNm=242.807   PNm=242.807              ,
                      RNm=9.834     RNm=9.834     RNm=9.834     RNm=9.834     RNm=9.834                ,
                      PT=242.807    PT=242.807    PT=242.807    PT=242.807    PT=242.807               ,
                      RT=9.834      RT=9.834      RT=9.834      RT=9.834      RT=9.834                 ,
```

```
S_ =

                      2             3              4              5              6              R_star_N=18.4604    (index "2" :6) ,
                      PN=276.5732   PN=0           PN=0           PN=0           PN=0           PT=305.8248
[260.00 , 325.00)  :  RN=5.118      RN=0           RN=0           RN=0           RN=0                      ,
                      PNm=0         PNm=305.8248   PNm=305.8248   PNm=305.8248   PNm=305.8248              ,
                      RNm=0         RNm=18.4604    RNm=18.4604    RNm=18.4604    RNm=18.4604               ,
                      PT=276.5732   PT=305.8248    PT=305.8248    PT=305.8248    PT=305.8248               ,
                      RT=5.118      RT=18.4604     RT=18.4604     RT=18.4604     RT=18.4604                ,
```

```
S_ =

                          2              3              4              5              6                R_star_N=21.3538    (index "2" :7) ,
                          PN=0           PN=0           PN=0           PN=0           PN=0             PT=356.7956
[325.00 , 390.00)  :      RN=0           RN=0           RN=0           RN=0           RN=0                                ,
                          PNm=356.7956   PNm=356.7956   PNm=356.7956   PNm=356.7956   PNm=356.7956                        ,
                          RNm=21.3538    RNm=21.3538    RNm=21.3538    RNm=21.3538    RNm=21.3538                         ,
                          PT=356.7956    PT=356.7956    PT=356.7956    PT=356.7956    PT=356.7956                         ,
                          RT=21.3538     RT=21.3538     RT=21.3538     RT=21.3538     RT=21.3538                          ,
```

```
S_ =

                          2              3              4              5              6                R_star_N=24.2472    (index "2" :8) ,
                          PN=0           PN=414.8598    PN=0           PN=0           PN=0             PT=407.7664
[390.00 , 455.00)  :      RN=0           RN=7.127       RN=0           RN=0           RN=0                                ,
                          PNm=407.7664   PNm=0          PNm=407.7664   PNm=407.7664   PNm=407.7664                        ,
                          RNm=24.2472    RNm=0          RNm=24.2472    RNm=24.2472    RNm=24.2472                         ,
                          PT=407.7664    PT=414.8598    PT=407.7664    PT=407.7664    PT=407.7664                         ,
                          RT=24.2472     RT=7.127       RT=24.2472     RT=24.2472     RT=24.2472                          ,
```

```
S_ =

                          2              3              4              5              6                R_star_N=30.034    (index "2" :10) ,
                          PN=276.5732    PN=0           PN=0           PN=0           PN=0             PT=509.708
[455.00 , 520.00)  :      RN=5.118       RN=0           RN=0           RN=0           RN=0                                ,
                          PNm=242.807    PNm=509.708    PNm=509.708    PNm=509.708    PNm=509.708                         ,
                          RNm=9.834      RNm=30.034     RNm=30.034     RNm=30.034     RNm=30.034                          ,
                          PT=519.3802    PT=509.708     PT=509.708     PT=509.708     PT=509.708                          ,
                          RT=14.952      RT=30.034      RT=30.034      RT=30.034      RT=30.034                           ,
```

```
S_ =

                          2              3              4              5              6                R_star_N=32.9274    (index "2" :11) ,
                          PN=276.5732    PN=0           PN=553.1464    PN=0           PN=0             PT=560.6788
[520.00 , 585.00)  :      RN=5.118       RN=0           RN=9.136       RN=0           RN=0                                ,
                          PNm=305.8248   PNm=560.6788   PNm=0          PNm=560.6788   PNm=560.6788                        ,
                          RNm=18.4604    RNm=32.9274    RNm=0          RNm=32.9274    RNm=32.9274                         ,
                          PT=582.398     PT=560.6788    PT=553.1464    PT=560.6788    PT=560.6788                         ,
                          RT=23.5784     RT=32.9274     RT=9.136       RT=32.9274     RT=32.9274                          ,
```

```
S_ =

                          2              3              4              5              6                R_star_N=32.9346    (index "2" :7) ,(index "4" :6) ,
                          PN=276.5732    PN=0           PN=0           PN=0           PN=0             PT=648.164
[585.00 , 650.00)  :      RN=5.118       RN=0           RN=0           RN=0           RN=0                                ,
                          PNm=356.7956   PNm=648.164    PNm=648.164    PNm=648.164    PNm=648.164                         ,
                          RNm=21.3538    RNm=32.9346    RNm=32.9346    RNm=32.9346    RNm=32.9346                         ,
                          PT=633.3688    PT=648.164     PT=648.164     PT=648.164     PT=648.164                          ,
                          RT=26.4718     RT=32.9346     RT=32.9346     RT=32.9346     RT=32.9346                          ,
```
