# Supplementary material for: Constrained portfolio optimization with discrete variables: An algorithmic method based on dynamic programming
Source: PLoS One. 2022 Jul 28;17(7):e0271811. doi: 10.1371/journal.pone.0271811 (PMC9333297; doi:10.1371/journal.pone.0271811)
Supplement: S6 Appendix — (PDF) [file pone.0271811.s006.pdf]

Table 1: The manually solution for the fifth environmental share.

S	5	6	7	8	9	10	$F^*(5,S)$	X_5^*
[0,65)	0	0	0	0	0	0	0	-
[65, 130)	0	0	0	0	0	0	0	-
[130, 195)	0	0	0	0	0	0	0	-
[195, 260)	0	0	0	0	0	0	0	-
[260, 325)	P_5=287.5965<br>R_5=8.4965	0	0	0	0	0	R^*_5=8.4965<br>If<br>P_5=287.5965	5
[325, 390)	0	P_5=345.1158<br>R_5=9.9758	0	0	0	0	R^*_5=9.9758<br>If<br>P_5=345.1158	6
[390, 455)	0	0	P_5= 402.6351<br>R_5= 11.4551	0	0	0	R^*_5=11.4551<br>If<br>P_5=402.6351	7
[455, 520)	0	0	0	P_5=460.1544<br>R_5=12.9344	P_5=517.6737<br>R_5=14.4137	0	R^*_5=14.4137<br>If<br>P_5=517.6737	9
[520, 585)	0	0	0	0	0	P_5=575.193<br>R_5=15.893	R^*_5=15.893<br>If<br>P_5=575.193	10
[585, 650]	0	0	0	-	0	0	0	-

Table 2: The manually solution for the fourth environmental share.

S	5	6	7	8	9	10	11	$F^*(4,S)$	X_4^*
[0,65)	0	0	0	0	0	0	0	0	-
[65, 130)	0	0	0	0	0	0	0	0	-
[130, 195)	0	0	0	0	0	0	0	0	-
[195, 260)	P_4= 242.807<br>R_4= 9.834<br>R_5= 0<br>P_5=0<br>P_T= 242.807<br>R_T=12.834	0	0	0	0	0	0	R^*_4=9.834<br>If<br>P_T= 242.807	$X_4^* = 5$<br>$X_5^* = -$
[260, 325)	R_4= 0<br>P_4=0<br>P_5=287.5965<br>R_5=8.4965<br>P_T= 287.5965<br>R_T=8.4965	P_4= 291.3684<br>R_4= 11.58.08<br>R_5= 0<br>P_5=0<br>P_T= 291.3684<br>R_T=11.58.08	R_4= 0<br>P_4=0<br>P_5=287.5965<br>R_5=8.4965<br>P_T= 287.5965<br>R_T=8.4965	R_4= 0<br>P_4=0<br>P_5=287.5965<br>R_5=8.4965<br>P_T= 287.5965<br>R_T=8.4965	R_4= 0<br>P_4=0<br>P_5=287.5965<br>R_5=8.4965<br>P_T= 287.5965<br>R_T=8.4965	R_4= 0<br>P_4=0<br>P_5=287.5965<br>R_5=8.4965<br>P_T= 287.5965<br>R_T=8.4965	R_4= 0<br>P_4=0<br>P_5=287.5965<br>R_5=8.4965<br>P_T= 287.5965<br>R_T=8.4965	R^*_4=11.5808<br>If<br>P_T= 291.3684	$X_4^* = 6$<br>$X_5^* = -$
[325, 390)	R_4= 0<br>P_4=0<br>P_5=345.1158<br>R_5=9.9758<br>P_T= 345.1158<br>R_T=9.9758	R_4= 0<br>P_4=0<br>P_5=345.1158<br>R_5=9.9758<br>P_T= 345.1158<br>R_T=9.9758	P_4= 339.9298<br>R_4= 13.3276<br>R_5= 0<br>P_5=0<br>P_T= 339.9298<br>R_T=13.3276	P_4= 388.4912<br>R_4= 13.0744<br>R_5= 0<br>P_5=0<br>P_T= 388.4912<br>R_T=15.0744	R_4= 0<br>P_4=0<br>P_5=345.1158<br>R_5=9.9758<br>P_T= 345.1158<br>R_T=9.9758	R_4= 0<br>P_4=0<br>P_5=345.1158<br>R_5=9.9758<br>P_T= 345.1158<br>R_T=9.9758	R_4= 0<br>P_4=0<br>P_5=345.1158<br>R_5=9.9758<br>P_T= 345.1158<br>R_T=9.9758	R^*_4=15.0744<br>If<br>P_T= 388.4912	$X_4^* = 8$<br>$X_5^* = -$
[390, 455)	R_4= 0<br>P_4=0<br>P_5= 402.6351<br>R_5= 11.4551<br>P_T= 402.6351<br>R_T=11.4551	R_4= 0<br>P_4=0<br>P_5= 402.6351<br>R_5= 11.4551<br>P_T= 402.6351<br>R_T=11.4551	R_4= 0<br>P_4=0<br>P_5= 402.6351<br>R_5= 11.4551<br>P_T= 402.6351<br>R_T=11.4551	R_4= 0<br>P_4=0<br>P_5= 402.6351<br>R_5= 11.4551<br>P_T= 402.6351<br>R_T=11.4551	P_4= 437.0526<br>R_4= 16.8212<br>R_5= 0<br>P_5=0<br>P_T= 437.0526<br>R_T= 16.8212	R_4= 0<br>P_4=0<br>P_5= 402.6351<br>R_5= 11.4551<br>P_T= 402.6351<br>R_T=11.4551	R_4= 0<br>P_4=0<br>P_5= 402.6351<br>R_5= 11.4551<br>P_T= 402.6351<br>R_T=11.4551	R^*_4= 16.8212<br>If<br>P_T= 437.0526	$X_4^* = 9$<br>$X_5^* = -$
[455, 520)	R_4= 0<br>P_4=0<br>P_5=517.6337<br>R_5=14.4137<br>P_T= 517.6337<br>R_T=14.4137	R_4= 0<br>P_4=0<br>P_5=517.6337<br>R_5=14.4137<br>P_T= 517.6337<br>R_T=14.4137	R_4= 0<br>P_4=0<br>P_5=517.6337<br>R_5=14.4137<br>P_T= 517.6337<br>R_T= 14.4137	R_4= 0<br>P_4=0<br>P_5=517.6337<br>R_5=14.4137<br>P_T= 517.6337<br>R_T= 14.4137	R_4= 0<br>P_4=0<br>P_5=517.6337<br>R_5=14.4137<br>P_T= 517.6337<br>R_T= 14.4137	P_4= 485.614<br>R_4= 18.568<br>R_5= 0<br>P_5=0<br>P_T= 485.614<br>R_T= 18.568	R_4= 0<br>P_4=0<br>P_5=517.6337<br>R_5=14.4137<br>P_T= 517.6337<br>R_T= 14.4137	R^*_4= 18.568<br>If<br>P_T= 485.614	$X_4^* = 10$<br>$X_5^* = -$
[520, 585)	P_4= 242.807<br>R_4= 9.834<br>P_5=287.5965<br>R_5=8.4965<br>P_T= 530.4035<br>R_T=18.3305	P_4= 291.3684<br>R_4= 11.58.08<br>P_5=287.5965<br>R_5=8.4965<br>P_T= 578.9649<br>R_T=20.0773	R_4= 0<br>P_4=0<br>P_5=575.193<br>R_5=15.893<br>P_T= 575.193<br>R_T=15.893	R_4= 0<br>P_4=0<br>P_5=575.193<br>R_5=15.893<br>P_T= 575.193<br>R_T=15.893	R_4= 0<br>P_4=0<br>P_5=575.193<br>R_5=15.893<br>P_T= 575.193<br>R_T=15.893	R_4= 0<br>P_4=0<br>P_5=575.193<br>R_5=15.893<br>P_T= 575.193<br>R_T=15.893	P_4= 534.1761<br>R_4= 20.3148<br>R_5= 0<br>P_5=0<br>P_T= 534.1761<br>R_T= 20.3148	R^*_4=20.3148<br>If<br>P_T= 534.1761	$X_4^* = 11$<br>$X_5^* = -$
[585, 650]	P_4= 242.807<br>R_4= 9.834<br>P_5= 402.6351<br>R_5= 11.4551<br>P_T= 645.4421<br>R_T=21.2891	P_4= 291.3684<br>R_4= 11.58.08<br>P_5=345.1158<br>R_5=9.9758<br>P_T= 636.4842<br>R_T=21.5566	P_4= 339.9298<br>R_4= 13.3276<br>P_5=287.5965<br>R_5=8.4965<br>P_T= 627.5263<br>R_T= 21.8241	0	0	0	0	R^*_4= 21.8241<br>If<br>P_T= 627.5263	$X_4^* = 7$<br>$X_5^* = 5$

Table 3: The manually solution for the third environmental share.

S	5	6	7	8	9	$F^*(3,S)$	X_3^*
[0,65)	0	0	0	0	0	0	-
[65, 130)	0	0	0	0	0	0	-
[130, 195)	0	0	0	0	0	0	-
[195, 260)	R_3= 0<br>P_3=0<br>P_4= 242.807<br>R^*_4= 9.834<br>P_T= 242.807<br>R_T=9.834	R_3= 0<br>P_3=0<br>P_4= 242.807<br>R^*_4= 9.834<br>P_T= 242.807<br>R_T=9.834	R_3= 0<br>P_3=0<br>P_4= 242.807<br>R^*_4= 9.834<br>P_T= 242.807<br>R_T= 9.834	R_3= 0<br>P_3=0<br>P_4= 242.807<br>R^*_4= 9.834<br>P_T= 242.807<br>R_T= 9.834	R_3= 0<br>P_3=0<br>P_4= 242.807<br>R^*_4= 9.834<br>P_T= 242.807<br>R_T= 9.834	R^*_3=9.834<br>If<br>P_T= 242.807	$X_3^* = -$<br>$X_4^* = 5$<br>$X_5^* = -$
[260, 325)	R_3= 0<br>P_3=0<br>P_4= 291.3684<br>R^*_4= 11.58.08<br>P_T= 291.3684<br>R_T=11.58.08	R_3= 0<br>P_3=0<br>P_4= 291.3684<br>R^*_4= 11.58.08<br>P_T= 291.3684<br>R_T=11.58.08	R_3= 0<br>P_3=0<br>P_4= 291.3684<br>R^*_4= 11.58.08<br>P_T= 291.3684<br>R_T=11.58.08	R_3= 0<br>P_3=0<br>P_4= 291.3684<br>R^*_4= 11.58.08<br>P_T= 291.3684<br>R_T=11.58.08	R_3= 0<br>P_3=0<br>P_4= 291.3684<br>R^*_4= 11.58.08<br>P_T= 291.3684<br>R_T=11.58.08	R^*_3=11.5808<br>If<br>P_T= 291.3684	$X_3^* = -$<br>$X_4^* = 6$<br>$X_5^* = -$
[325, 390)	P_3= 362.8535<br>R_3= 17.8975<br>R_4= 0<br>P_4= 0<br>P_T= 362.8535<br>R_T= 17.8975	R_3= 0<br>P_3=0<br>P_4= 388.4912<br>R^*_4= 15.0744<br>P_T= 388.4912<br>R_T=15.0744	R_3= 0<br>P_3=0<br>P_4= 388.4912<br>R^*_4= 15.0744<br>P_T= 388.4912<br>R_T=15.0744	R_3= 0<br>P_3=0<br>P_4= 388.4912<br>R^*_4= 15.0744<br>P_T= 388.4912<br>R_T=15.0744	R_3= 0<br>P_3=0<br>P_4= 388.4912<br>R^*_4= 15.0744<br>P_T= 388.4912<br>R_T=15.0744	R^*_3= 17.8975<br>If<br>P_T= 362.8535	$X_3^* = 5$<br>$X_4^* = -$<br>$X_5^* = -$
[390, 455)	R_3= 0<br>P_3=0<br>P_4= 437.0526<br>R^*_4= 16.8212<br>P_T= 437.0526<br>R_T= 16.8212	P_3= 435.4242<br>R_3= 21.257<br>R_4= 0<br>P_4= 0<br>P_T= 435.4242<br>R_T= 21.257	R_3= 0<br>P_3=0<br>P_4= 437.0526<br>R^*_4= 16.8212<br>P_T= 437.0526<br>R_T= 16.8212	R_3= 0<br>P_3=0<br>P_4= 437.0526<br>R^*_4= 16.8212<br>P_T= 437.0526<br>R_T= 16.8212	R_3= 0<br>P_3=0<br>P_4= 437.0526<br>R^*_4= 16.8212<br>P_T= 437.0526<br>R_T= 16.8212	R^*_3= 21.257<br>If<br>P_T= 435.4242	$X_3^* = 6$<br>$X_4^* = -$<br>$X_5^* = -$
[455, 520)	R_3= 0<br>P_3=0<br>P_4= 485.614<br>R^*_4= 18.568<br>P_T= 485.614<br>R_T= 18.568	R_3= 0<br>P_3=0<br>P_4= 485.614<br>R^*_4= 18.568<br>P_T= 485.614<br>R_T= 18.568	P_3=507.9949<br>R_3= 24.6165<br>R_4= 0<br>P_4= 0<br>P_T=507.9949<br>R_T=24.6165	R_3= 0<br>P_3=0<br>P_4= 485.614<br>R^*_4= 18.568<br>P_T= 485.614<br>R_T= 18.568	R_3= 0<br>P_3=0<br>P_4= 485.614<br>R^*_4= 18.568<br>P_T= 485.614<br>R_T= 18.568	R^*_3=24.6165<br>If<br>P_T=507.9949	$X_3^* = 7$<br>$X_4^* = -$<br>$X_5^* = -$
[520, 585)	R_3= 0<br>P_3=0<br>R^*_4=20.3148<br>P_4= 534.1754<br>P_T= 534.1754<br>R_T=20.3148	R_3= 0<br>P_3=0<br>R^*_4=20.3148<br>P_4= 534.1754<br>P_T= 534.1754<br>R_T=20.3148	R_3= 0<br>P_3=0<br>R^*_4=20.3148<br>P_4= 534.1754<br>P_T= 534.1754<br>R_T=20.3148	P_3=580.5656<br>R_3= 27.976<br>R_4= 0<br>P_4= 0<br>P_T= 580.5656<br>R_T= 27.976	R_3= 0<br>P_3=0<br>R^*_4=20.3148<br>P_4= 534.1754<br>P_T= 534.1754<br>R_T=20.3148	R^*_3= 27.976<br>If<br>P_T= 580.5656	$X_3^* = 8$<br>$X_4^* = -$<br>$X_5^* = -$
[585, 650]	P_3= 362.8535<br>R_3= 17.8975<br>P_4= 242.807<br>R^*_4= 9.834<br>P_T= 605.6605<br>R_T= 27.7315	R_3= 0<br>P_3=0<br>P_4= 627.5263<br>R^*_4= 21.8241<br>P_T= 627.5263<br>R_T= 21.8241	R_3= 0<br>P_3=0<br>P_4= 627.5263<br>R^*_4= 21.8241<br>P_T= 627.5263<br>R_T= 21.8241	R_3= 0<br>P_3=0<br>P_4= 627.5263<br>R^*_4= 21.8241<br>P_T= 627.5263<br>R_T= 21.8241	R_3= 0<br>P_3=0<br>P_4= 627.5263<br>R^*_4= 21.8241<br>P_T= 627.5263<br>R_T= 21.8241	R^*_3= 27.7315<br>If<br>P_T= 605.6605	$X_3^* = 5$<br>$X_4^* = 5$<br>$X_5^* = -$

Table 4: The manually solution for the second environmental share.

S	6	7	8	9	10	11	$F^*(2,S)$	X_2^*
[0,65)	0	0	0	0	0	0	0	-
[65, 130)	0	0	0	0	0	0	0	-
[130, 195)	0	0	0	0	0	0	0	-
[195, 260)	R_2=0<br>P_2= 0<br>R^*_3=9.834<br>P_3= 242.807<br>P_T= 242.807<br>R_T=9.834	R_2=0<br>P_2= 0<br>R^*_3=9.834<br>P_3= 242.807<br>P_T= 242.807<br>R_T=9.834	R_2=0<br>P_2= 0<br>R^*_3=9.834<br>P_3= 242.807<br>P_T= 242.807<br>R_T=9.834	R_2=0<br>P_2= 0<br>R^*_3=9.834<br>P_3= 242.807<br>P_T= 242.807<br>R_T=9.834	R_2=0<br>P_2= 0<br>R^*_3=9.834<br>P_3= 242.807<br>P_T= 242.807<br>R_T=9.834	R_2=0<br>P_2= 0<br>R^*_3=9.834<br>P_3= 242.807<br>P_T= 242.807<br>R_T=9.834	R^*_2=9.834<br>If<br>P_T= 242.807	$X_2^* = -$<br>$X_3^* = -$<br>$X_4^* = 5$<br>$X_5^* = -$
[260, 325)	P_2= 305.8248<br>R_2= 18.4604<br>R^*_3= 0<br>P_3= 0<br>P_T= 305.8248<br>R_T= 18.4604	R_2=0<br>P_2= 0<br>R^*_3=11.5808<br>P_3= 291.3684<br>P_T= 291.3684<br>R_T=11.5808	R_2=0<br>P_2= 0<br>R^*_3=11.5808<br>P_3= 291.3684<br>P_T= 291.3684<br>R_T=11.5808	R_2=0<br>P_2= 0<br>R^*_3=11.5808<br>P_3= 291.3684<br>P_T= 291.3684<br>R_T=11.5808	R_2=0<br>P_2= 0<br>R^*_3=11.5808<br>P_3= 291.3684<br>P_T= 291.3684<br>R_T=11.5808	R_2=0<br>P_2= 0<br>R^*_3=11.5808<br>P_3= 291.3684<br>P_T= 291.3684<br>R_T=11.5808	R^*_2= 18.4604<br>If<br>P_T= 305.8248	$X_2^* = 6$<br>$X_3^* = -$<br>$X_4^* = -$<br>$X_5^* = -$
[325, 390)	R_2=0<br>P_2= 0<br>R^*_3= 17.8975<br>P_3= 362.8535<br>P_T= 362.8535<br>R_T= 17.8975	P_2= 356.7956<br>R_2= 21.3538<br>R^*_3= 0<br>P_3= 0<br>P_T= 356.7956<br>R_T= 21.3538	R_2=0<br>P_2= 0<br>R^*_3= 17.8975<br>P_3= 362.8535<br>P_T= 362.8535<br>R_T= 17.8975	R_2=0<br>P_2= 0<br>R^*_3= 17.8975<br>P_3= 362.8535<br>P_T= 362.8535<br>R_T= 17.8975	R_2=0<br>P_2= 0<br>R^*_3= 17.8975<br>P_3= 362.8535<br>P_T= 362.8535<br>R_T= 17.8975	R_2=0<br>P_2= 0<br>R^*_3= 17.8975<br>P_3= 362.8535<br>P_T= 362.8535<br>R_T= 17.8975	R^*_2= 21.3538<br>If<br>P_T= 356.7956	$X_2^* = 7$<br>$X_3^* = -$<br>$X_4^* = -$<br>$X_5^* = -$
[390, 455)	R_2=0<br>P_2= 0<br>R^*_3= 21.257<br>P_3= 435.4242<br>P_T= 435.4242<br>R_T= 21.257	R_2=0<br>P_2= 0<br>R^*_3= 21.257<br>P_3= 435.4242<br>P_T= 435.4242<br>R_T= 21.257	P_2= 407.7664<br>R_2= 24.2472<br>R^*_3= 0<br>P_3= 0<br>P_T= 407.7664<br>R_T= 24.2472	R_2=0<br>P_2= 0<br>R^*_3= 21.257<br>P_3= 435.4242<br>P_T= 435.4242<br>R_T= 21.257	R_2=0<br>P_2= 0<br>R^*_3= 21.257<br>P_3= 435.4242<br>P_T= 435.4242<br>R_T= 21.257	R_2=0<br>P_2= 0<br>R^*_3= 21.257<br>P_3= 435.4242<br>P_T= 435.4242<br>R_T= 21.257	R^*_2= 27.2472<br>If<br>P_T= 407.7664	$X_2^* = 8$<br>$X_3^* = -$<br>$X_4^* = -$<br>$X_5^* = -$
[455, 520)	R_2=0<br>P_2= 0<br>R^*_3=24.6165<br>P_3=507.9949<br>P_T= 507.9949<br>R_T= 24.6165	R_2=0<br>P_2= 0<br>R^*_3=24.6165<br>P_3=507.9949<br>P_T= 507.9949<br>R_T= 24.6165	R_2=0<br>P_2= 0<br>R^*_3=24.6165<br>P_3=507.9949<br>P_T= 507.9949<br>R_T= 24.6165	P_2= 458.7372<br>R_2= 27.1406<br>R^*_3= 0<br>P_3= 0<br>P_T= 458.7372<br>R_T= 27.1406	P_2= 509.708<br>R_2= 30.034<br>R^*_3= 0<br>P_3= 0<br>P_T= 509.708<br>R_T= 30.034	R_2=0<br>P_2= 0<br>R^*_3=24.6165<br>P_3=507.9949<br>P_T= 507.9949<br>R_T= 24.6165	R^*_2= 30.034<br>If<br>P_T= 509.708	$X_2^* = 10$<br>$X_3^* = -$<br>$X_4^* = -$<br>$X_5^* = -$
[520, 585)	P_2= 305.8248<br>R_2= 18.4604<br>R^*_3=9.834<br>P_3= 242.807<br>P_T= 548.6318<br>R_T= 28.2944	R_2=0<br>P_2= 0<br>R^*_3= 27.976<br>P_3= 580.5656<br>P_T= 580.5656<br>R_T= 27.976	R_2=0<br>P_2= 0<br>R^*_3= 27.976<br>P_3= 580.5656<br>P_T= 580.5656<br>R_T= 27.976	R_2=0<br>P_2= 0<br>R^*_3=27.976<br>P_3= 580.5656<br>P_T= 580.5656<br>R_T= 27.976	R_2=0<br>P_2= 0<br>R^*_3= 27.976<br>P_3= 580.5656<br>P_T= 580.5656<br>R_T= 27.976	P_2= 560.6788<br>R_2= 32.9274<br>R^*_3= 0<br>P_3= 0<br>P_T= 560.6788<br>R_T= 32.9274	R^*_2= 32.9274<br>If<br>P_T= 560.6788	$X_2^* = 11$<br>$X_3^* = -$<br>$X_4^* = -$<br>$X_5^* = -$
[585, 650]	P_2= 305.8248<br>R_2= 18.4604<br>R^*_3=11.5808<br>P_3= 291.3684<br>P_T= 597.1932<br>R_T= 30.0412	P_2= 356.7956<br>R_2= 21.3538<br>R^*_3=11.5808<br>P_3= 291.3684<br>P_T= 648.164<br>R_T=32.9346	R_2=0<br>P_2= 0<br>R^*_3= 27.7315<br>P_3= 605.6605<br>P_T= 605.6605<br>R_T= 27.7315	R_2=0<br>P_2= 0<br>R^*_3= 27.7315<br>P_3= 605.6605<br>P_T= 605.6605<br>R_T= 27.7315	R_2=0<br>P_2= 0<br>R^*_3= 27.7315<br>P_3= 605.6605<br>P_T= 605.6605<br>R_T= 27.7315	R_2=0<br>P_2= 0<br>R^*_3= 27.7315<br>P_3= 605.6605<br>P_T= 605.6605<br>R_T= 27.7315	R^*_2= 32.9346<br>If<br>P_T= 648.164	$X_2^* = 7$<br>$X_3^* = -$<br>$X_4^* = 6$<br>$X_5^* = -$

Table 5: The manually solution for the first environmental share.

S	2	3	4	5	6	$F^*(1,S)$	X_1^*
[0,65)	0	0	0	0	0	0	-
[65, 130)	0	0	0	0	0	0	-
[130, 195)	0	0	0	0	0	0	-
[195, 260)	R_1=0<br>P_1= 0<br>R^*_2=9.834<br>P_2= 242.807<br>P_T= 242.807<br>R_T= 9.834	R_1=0<br>P_1= 0<br>R^*_2=9.834<br>P_2= 242.807<br>P_T= 242.807<br>R_T= 9.834	R_1=0<br>P_1= 0<br>R^*_2=9.834<br>P_2= 242.807<br>P_T= 242.807<br>R_T= 9.834	R_1=0<br>P_1= 0<br>R^*_2=9.834<br>P_2= 242.807<br>P_T= 242.807<br>R_T= 9.834	R_1=0<br>P_1= 0<br>R^*_2=9.834<br>P_2= 242.807<br>P_T= 242.807<br>R_T= 9.834	R^*_1=9.834<br>If<br>P_T= 242.807	$X_1^* = -$<br>$X_2^* = -$<br>$X_3^* = -$<br>$X_4^* = 5$<br>$X_5^* = -$
[260, 325)	P_1= 276.5732<br>R_1= 5.1018<br>R^*_2= 0<br>P_2= 0<br>P_T= 276.5732<br>R_T= 5.1018	R_1=0<br>P_1= 0<br>R^*_2= 18.4604<br>P_2= 305.8248<br>P_T= 305.8248<br>R_T=18.4604	R_1=0<br>P_1= 0<br>R^*_2= 18.4604<br>P_2= 305.8248<br>P_T= 305.8248<br>R_T=18.4604	R_1=0<br>P_1= 0<br>R^*_2= 18.4604<br>P_2= 305.8248<br>P_T= 305.8248<br>R_T=18.4604	R_1=0<br>P_1= 0<br>R^*_2= 18.4604<br>P_2= 305.8248<br>P_T= 305.8248<br>R_T=18.4604	R^*_1=18.4604<br>If<br>P_T= 305.8248	$X_1^* = -$<br>$X_2^* = 6$<br>$X_3^* = -$<br>$X_4^* = -$<br>$X_5^* = -$
[325, 390)	R_1=0<br>P_1= 0<br>R^*_2= 21.3538<br>P_2= 356.7956<br>P_T= 356.7956<br>R_T= 21.3538	R_1=0<br>P_1= 0<br>R^*_2= 21.3538<br>P_2= 356.7956<br>P_T= 356.7956<br>R_T= 21.3538	R_1=0<br>P_1= 0<br>R^*_2= 21.3538<br>P_2= 356.7956<br>P_T= 356.7956<br>R_T= 21.3538	R_1=0<br>P_1= 0<br>R^*_2= 21.3538<br>P_2= 356.7956<br>P_T= 356.7956<br>R_T= 21.3538	R_1=0<br>P_1= 0<br>R^*_2= 21.3538<br>P_2= 356.7956<br>P_T= 356.7956<br>R_T= 21.3538	R^*_1= 21.3538<br>If<br>P_T= 356.7956	$X_1^* = -$<br>$X_2^* = 7$<br>$X_3^* = -$<br>$X_4^* = -$<br>$X_5^* = -$
[390, 455)	R_1=0<br>P_1= 0<br>R^*_2= 24.2472<br>P_2= 407.7664<br>P_T= 407.7664<br>R_T=24.2472	P_1= 414.8598<br>R_1= 7.1027<br>R^*_2= 0<br>P_2= 0<br>P_T= 414.8598<br>R_T= 7.1027	R_1=0<br>P_1= 0<br>R^*_2= 24.2472<br>P_2= 407.7664<br>P_T= 407.7664<br>R_T=24.2472	R_1=0<br>P_1= 0<br>R^*_2= 24.2472<br>P_2= 407.7664<br>P_T= 407.7664<br>R_T=24.2472	R_1=0<br>P_1= 0<br>R^*_2= 24.2472<br>P_2= 407.7664<br>P_T= 407.7664<br>R_T=24.2472	R^*_1=24.2472<br>If<br>P_T= 407.7664	$X_1^* = -$<br>$X_2^* = 8$<br>$X_3^* = -$<br>$X_4^* = -$<br>$X_5^* = -$
[455, 520)	P_1= 276.5732<br>R_1= 5.1018<br>R^*_2=9.834<br>P_2= 242.807<br>P_T= 519.3802<br>R_T= 14.9358	R_1=0<br>P_1= 0<br>R^*_2= 30.034<br>P_2= 509.708<br>P_T= 509.708<br>R_T=30.034	R_1=0<br>P_1= 0<br>R^*_2= 30.034<br>P_2= 509.708<br>P_T= 509.708<br>R_T=30.034	R_1=0<br>P_1= 0<br>R^*_2= 30.034<br>P_2= 509.708<br>P_T= 509.708<br>R_T=30.034	R_1=0<br>P_1= 0<br>R^*_2= 30.034<br>P_2= 509.708<br>P_T= 509.708<br>R_T=30.034	R^*_1=30.034<br>If<br>P_T= 509.708	$X_1^* = -$<br>$X_2^* = 10$<br>$X_3^* = -$<br>$X_4^* = -$<br>$X_5^* = -$
[520, 585)	P_1= 276.5732<br>R_1= 5.1018<br>R^*_2= 18.4604<br>P_2= 305.8248<br>P_T= 582.398<br>R_T=23.5622	R_1=0<br>P_1= 0<br>R^*_2= 32.9274<br>P_2= 560.6788<br>P_T= 560.6788<br>R_T=32.9274	P_1= 553.1464<br>R_1= 9.1036<br>R^*_2= 0<br>P_2= 0<br>P_T= 553.1464<br>R_T= 9.1036	R_1=0<br>P_1= 0<br>R^*_2= 32.9274<br>P_2=560.6788<br>P_T= 560.6788<br>R_T=32.9274	R_1=0<br>P_1= 0<br>R^*_2= 32.9274<br>P_2= 560.6788<br>P_T= 560.6788<br>R_T=32.9274	R^*_1=32.9274<br>If<br>P_T= 560.6788	$X_1^* = -$<br>$X_2^* = 11$<br>$X_3^* = -$<br>$X_4^* = -$<br>$X_5^* = -$
[585, 650]	P_1= 276.5732<br>R_1= 5.1018<br>R^*_2= 21.3538<br>P_2= 356.7956<br>P_T= 633.3688<br>R_T= 26.4556	R_1=0<br>P_1= 0<br>R^*_2= 32.9346<br>P_2= 648.164<br>P_T= 648.164<br>R_T= 32.9346	R_1=0<br>P_1= 0<br>R^*_2= 32.9346<br>P_2= 648.164<br>P_T= 648.164<br>R_T= 32.9346	R_1=0<br>P_1= 0<br>R^*_2= 32.9346<br>P_2= 648.164<br>P_T= 648.164<br>R_T= 32.9346	R_1=0<br>P_1= 0<br>R^*_2= 32.9346<br>P_2= 648.164<br>P_T= 648.164<br>R_T= 32.9346	R^*_1= 32.9346<br>If<br>P_T= 648.164	$X_1^* = -$<br>$X_2^* = 7$<br>$X_3^* = -$<br>$X_4^* = 6$<br>$X_5^* = -$
