# Supplementary material for: Constrained portfolio optimization with discrete variables: An algorithmic method based on dynamic programming
Source: PLoS One. 2022 Jul 28;17(7):e0271811. doi: 10.1371/journal.pone.0271811 (PMC9333297; doi:10.1371/journal.pone.0271811)
Supplement: Attachment — Submitted filename: Response to reviewers.pdf [file pone.0271811.s007.pdf]

Dear editorial board and referees of PLOS ONE,

May 6, 2022

We appreciate the interest that the editors and reviewers have taken in our manuscript and the constructive criticism they have given. We have addressed the major concerns of the reviewers.

Please allow us to explain how we dealt with the issues raised in your letter. We have also attached separate responses to each of the reviewers.

Thank you again for consideration of our revised manuscript.

Best regards,

Fereshteh Vaezi Jezeie, PhD.
Department of Industrial Engineering.
Iran University of Science and Technology.
Address: Narmak, Tehran, Iran. P.O. Box 1684613114.
Phone Number: +989127185240.
fax: +982177240482.
E-mail: f.vaezi.92@gmail.com

Reviewer #1

This paper proposes a methodology for solving a portfolio optimization problem using a dynamic programming method. The manuscript has several structural drawbacks, some of which are as follows:

Reviewer Comment (1): The introduction section is not written in a proper condition. The contributions in this section are not adequately expressed. The difference between the proposed method and the previously published papers on applying the dynamic programming method to portfolio optimization problems is not discussed. Based on an author's claim, "The study aims to find a more efficient method for the portfolio optimization problems." The reasons for the superiority of the proposed method should be considered in the introduction section.

Answer (1): Thank you for your valuable comment. The issues have been corrected in the manuscript (line 43 to 52- line 87 to 105).

Reviewer Comment (2): Based on constraint (3), eq(7) is incorrect.

Answer (2): Thank you for your comment. We have added a cardinality constraint with a coefficient to the objective function. In fact, Eq. (3) is added to the objective function with the coefficient (λ) as a penalty. λ has a negative sign. λ has taken negative values in the case study.
Moreover, λ is determined based on K. The first step to solve the case study is to obtain the optimal value of λ (λ^*). λ^* is obtained from the sequential approximation method using the designed MATLAB code (Appendix 1) based on K. Moreover, the Intlinprog solver in MATLAB has also been used to check the accuracy and validity of the designed MATLAB code results (Appendix 2). According to the results, the first place where K in the cardinality constraint meets is where λ is λ^*. Moreover, all symbols and indices have been checked and corrected.

Reviewer Comment (3): In the first presentation of the portfolio optimization problem, each constraint is numbered, but this is not the case in the second statement of the problem.

Answer (3): Thank you for your detailed comment. The issues have been corrected in the manuscript (lines 129 to 130).

Reviewer Comment (4): The notation of eq(2) differed from the corresponding equation in Eq(8).

Answer (4): Thank you for your detailed comment. The issues have been corrected in the manuscript. All symbols and indices have been checked and corrected.

Reviewer Comment (5): The title "Dynamic programming" is a general topic that is unsuitable for a research paper.

Answer (5): Thank you for your detailed comment. We have tried to change the topic (line 2 to 3).

Reviewer Comment (6): This paper needs a flowchart.

Answer (6): Thank you for your valuable comment. We have designed a flowchart (line 188 to 195).

Reviewer Comment (7): The steps in algorithm 1 need a simple example to make following the method easier.

Answer (7): Thank you for your comment. To make following the proposed method easier, the first case study has been considered and all related information are presented in S3 Table. Appendix (6). The first case study has been selected in small dimensions to show the solution of the proposed method manually.

Reviewer Comment (8): The numerical result section should be improved critically. In the first step, different case studies should be defined clearly. The input parameters for each case are described in the tables, and appropriate references should be assigned to each section. Please make sure that all parameters are defined precisely. Finally, the results are discussed.

Answer (8): Thank you for your valuable comment. The issues have been corrected in the manuscript (line 198 to 207 - line 213 to 224 - line 238 to 241 - line 245 to 248).

Reviewer Comment (9): The achieved results should be compared with other methods, especially the evolutionary methods.

Answer (9): Thank you for your valuable comment. We have compared the average results of Discrete Firefly Algorithm (DFA) and Genetic Algorithm (GA) whit the average results of the proposed algorithm based on dynamic programming (line 260 to 294).

Reviewer #2

This topic is interesting and seems to be practical. However, the quality of the manuscript should be improved carefully and based on the following comments.

Reviewer Comment (1): The English and writing of the manuscript should be improved. It needs extensive editing; the choice of words, language, syntax, phrasing, punctuation should be thoroughly checked and revised.

Answer (1): Thank you for your comment. The issues have been checked in the manuscript

Reviewer Comment (2): To make the contribution of the paper more clear, authors should summarize the characteristics of their method in the section of the introduction.

Answer (2): Thank you for your valuable comment. The issues have been corrected in the manuscript (line 43 to 52- line 87 to 105).

Reviewer Comment (3): The literature review sections should be improved; the focus should be on a critical analysis of the gradual advancement, as well as the current level, of the state-of-the-art, with quantitative information on the time & space complexity, as well as on the accuracy obtained by each cited methodology. The advancement offered by each cited methodology should be made clear.

Answer (3): Thank you for your comment. A literature review on portfolio optimization based on the dynamic programming approach is often provided for classical portfolio selection models with continuous variables. The methodology of each cited source has been carefully presented.

Reviewer Comment (4): Please provide the information of all indices in front of each mathematical equation. For example: Z, S, x

Answer (4): Thank you for your detailed comment. The issues have been checked in the manuscript and all changes have been shown in blue color.

Reviewer Comment (5): The comparative part of this paper is very weak. The author should make a more

comparative study to compare the efficiency of the proposed techniques with well-known existing techniques. For this optimization problem, it is better to compare several algorithms.

Answer (5): Thank you for your valuable comment. We have compared the average results of Discrete Firefly Algorithm (DFA) and Genetic Algorithm (GA) whit the average results of the proposed algorithm based on dynamic programming (line 260 to 294).

Reviewer Comment (6): Section 4 is not available!! (In section 4, two case studies of the US stock exchange are analyzed)

Answer (6): Thank you for your detailed comment. The issues have been corrected in the manuscript.

Reviewer Comment (7): There are two sections 5 and in terms of numbering the sections of the article should be corrected.

Answer (7): Thank you for your detailed comment. The issues have been corrected in the manuscript.

Reviewer Comment (8): To better understand the algorithm, it is recommended to use a flowchart with the algorithm.

Answer (8): Thank you for your valuable comment. We have designed a flowchart.

Thank you again for your positive reactions to our manuscript and for your very constructive comments. We sincerely hope that you like the changes we have made to the manuscript.
